# PPAR *γ*/Nnat/NF-*κ*B Axis Involved in Promoting Effects of Adiponectin on Preadipocyte Differentiation

**DOI:** 10.1155/2019/5618023

**Published:** 2019-11-21

**Authors:** Wenkai Yang, Wenjin Yuan, Xinghua Peng, Meiling Wang, Jie Xiao, Cheng Wu, Lie Luo

**Affiliations:** ^1^Department of Cardiovascular Surgery, Affiliated Central People's Hospital of Zhanjiang of Guangdong Medical University, Zhanjiang 524045, China; ^2^Department of Cardiovascular Medicine, Ganzhou People's Hospital, Ganzhou 341000, China; ^3^Department of Cardio-Thoracic Surgery, Ganzhou People's Hospital, Ganzhou 341000, China

## Abstract

A previous study has demonstrated that adiponectin (APN) could promote preadipocyte differentiation, and the present study further explored its mechanism. 3T3-L1 cells were infected with adenovirus holding human adiponectin gene apM1 and mouse neuronatin (Nnat) shRNA and initiated differentiation while coculturing with mature adipocytes stimulated with LPS. After 8 days, preadipocyte differentiation was observed by Oil Red O staining. Real-time quantitative PCR was used to evaluate mRNA expression levels of monocyte chemoattractant protein-1 (MCP-1), interleukin- (IL-) 6, IL-8, and tumor necrosis factor *α* (TNF-*α*). The levels of reactive oxygen species (ROS), total antioxidant capacity (T-AOC), malondialdehyde (MDA), and superoxide dismutase (SOD) in 3T3-L1 cells were detected. Western blotting was done to quantify the protein expression levels of Nnat, peroxisome proliferator-activated receptor (PPAR) *γ*, p65, and inhibitor of nuclear factor *κ*B (I*κ*B) *α*. Results demonstrated that APN overexpression markedly increased preadipocyte differentiation; inhibited gene expression of MCP-1, IL-6, IL-8, and TNF-*α*; reduced ROS and MDA release; increased T-AOC and SOD levels; upregulated Nnat, PPAR *γ*, and I*κ*B *α* protein expressions; and downregulated p65 protein expression under LPS stimulation. However, the effects of APN were markedly attenuated when Nnat expression was knocked down. Taken together, the present study provided evidences that the effects of APN on promoting preadipocyte differentiation under inflammatory conditions via anti-inflammation and antioxidative stress may be regulated by the PPAR *γ*/Nnat/NF-*κ*B signaling pathway.

## 1. Introduction

Epicardial adipose tissue (EAT) is an adipose tissue located between the myocardium and the pericardium, surrounding and contacting directly the blood vessels of the heart. The paracrine and vascular effects resulting from the close contacting between the EAT and coronary artery make a great contribution to the occurrence and development of atherosclerosis [1, 2]. Imaging studies have treated EAT as a quantifiable risk marker of cerebrovascular diseases (CVDs) [3, 4]. Adipose tissue is a nonnegligible endocrine organ, which secretes various sorts of cytokines, called “adipocytokines,” such as tumor necrosis factor- (TNF-) *α*, interleukin-6 (IL-6), and adiponectin (APN) [5]. Studies showed that EAT could express inflammatory cytokines and chemokines as other adipose tissues [6]. Disorder of cytokines secreted by EAT would induce inflammation and especially atherosclerosis [7], and APN has a protective effect on atherosclerosis [8, 9]. However, the regulation mechanism of APN on atherosclerosis still needs to be further discussed.

Neuronatin (Nnat) is a new neurodevelopmentally related gene cloned in 1994 and initially confirmed to be selectively expressed in the brain of neonatal mammals [10]. Subsequently, its expression was also detected in various nonneural tissues such as exocrine, islets, and fat [11–13]. Studies have shown that diabetes, obesity, Lafora disease (LD), and retinal degeneration are all involving abnormal expression of Nnat [14]. They may be caused by Nnat-mediated Ca signal abnormalities, inflammatory responses, glucose exchange, and misfolding of Nnat [15]. In a preliminary study, we have detected Nnat expression in the EAT of coronary artery disease (CAD) patients and found that Nnat protein expression was significantly downregulated compared with no-CAD patients. The expression level of Nnat may be closely related to the regulation of EAT secretion. Therefore, we simulated an inflammatory physiological environment in the EAT of atherosclerosis patients via stimulating the mature adipocytes by lipopolysaccharide (LPS) and observed the role of Nnat in the effect of APN on promoting preadipocyte differentiation.

## 2. Materials and Methods

### 2.1. Reagents

LPS was purchased from Sigma-Aldrich (St. Louis, MO, USA) and diluted in pyrogen-free 0.9% saline. Insulin was obtained from Sigma-Aldrich (USA). TRIzol reagent was obtained from Invitrogen (Carlsbad, CA, USA). Anti-human APN, anti-Nnat, anti-peroxisome proliferator-activated receptor (PPAR) *γ*, anti-p65, and anti-inhibitor of nuclear factor *κ*B (I*κ*B) *α* were purchased from Abcam (Cambridge, MA, USA). Anti-*β*-actin antibody was purchased from Jiancheng Bioengineering Institute of Nanjing (Nanjing, JS, China). Reactive oxygen species (ROS), total antioxidant capacity (T-AOC), malondialdehyde (MDA), and superoxide dismutase (SOD) detection kits were purchased from Jiancheng Bioengineering Institute of Nanjing (Nanjing, JS, China).

### 2.2. Cell Culture and Differentiation

3T3-L1 preadipocyte (Type Culture Collection of the Chinese Academy of Sciences, Shanghai, China) was induced with medium containing DMEM, 10% FBS, 0.5 mM 3-isobutyl-1-methylaxanthine, 0.5 mM dexamethasone, and 5 mg/ml insulin (MDI; Sigma-Aldrich; USA). The above medium was replaced with DMEM containing 10% FBS and 5 mg/ml insulin after 48 h (day 2) MDI induction. Then the medium was replaced every other day with DMEM dissolved with 10% FBS until day 8. The 3T3-L1 cells would differentiate to mature adipocytes on the 8th day, which was rounded up and filled with countless oil droplets [16].

### 2.3. Construction of Recombinant Adenovirus

#### 2.3.1. Recombinant Adenovirus Construction for Expressing Human Adiponectin

Human adiponectin gene apM1 (Ad-apM1) was cloned from human adipose to construct recombinant adenovirus as previously discussed [17].

#### 2.3.2. Recombinant Adenovirus Construction for Silencing Mouse Nnat Gene

**Figure 1:**
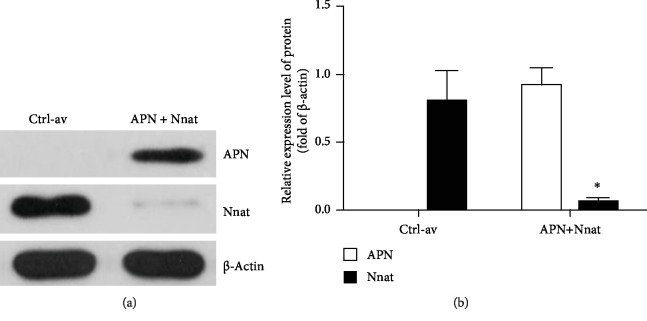
Expression levels of APN and Nnat following coinfection of 3T3-L1 cells with adenovirus holding Ad-apM1 and Ad-nnat-shRNA. For evaluating the compatibility of overexpression of 3T3-L1 mouse cells expressing the Ad-apM1 and Ad-nnat-shRNA, western blotting was used to detect the expression levels of Nnat and APN in 3T3-L1 cells after coinfecting with adenoviruses, respectively, holding Ad-apM1 and Ad-nnat-shRNA (APN + Nnat), or adenoviruses holding empty plasmids (Ctrl-av) only for 48 h. (a) Expression levels of human APN and mouse Nnat in groups of Ctrl-av and APN+Nnat. (b) The relative band intensity of APN and Nnat in each group. Data are presented as the mean ± standard error of the mean for six independent experiments. ^∗^*p* < 0.05 vs. the Ctrl-av group.

According to the design principle of shRNA (short hairpin RNA), the Nnat open reading frame region was selected as the interference target sequence (Ad-nnat-shRNA) and assembled to pAxCAwt holding U6 promoter (Cyagen Biosciences, Guangzhou, China), then cotransfecting into 293 cells using Lipofectamine 2000 (Invitrogen; Thermo Fisher Scientific, Inc.) following the manufacturer's instructions. Homologous recombination was used to produce recombinant adenovirus. In 293 cells, viruses were largely amplified. The sample was centrifuged for 20 min at 3,960 × g at 4°C after 48 h, and the supernatant was collected. Virus particles were concentrated by ultraspeed centrifugation (Beckman, Fullerton, CA, USA) at 5,000 × g for 2.5 h. The virus was packed and stored at -80°C. A tissue culture infective dose of 50 was used to detect virus titer. Western blotting was performed to verify protein expression levels of Nnat and APN following infection of 3T3-L1 cells with the adenovirus, and the results are shown in Figure 1.

### 2.4. Experimental Groups and Treatments

3T3-L1 cells (1 × 10^5^ cells), in the inner chamber of a 6-well Transwell plate (Corning, Corning, NY, USA), were cocultured with mature adipocytes (2 × 10^5^ cells) in the outer chamber, which were differentiated from 3T3-L1 cells. There were five different experimental groups as follows: (1) the control group (control), in which the 3T3-L1 cells in the inner chamber were induced differentiating into adipocytes as described above with mature adipocytes cultured in the outer chamber; (2) the LPS stimulation group (LPS), in which the mature adipocytes in the outer chamber were induced with inflammation by LPS (1 *μ*g/ml, 18 h) as previously discussed [18]. 3T3-L1 cells in the inner chamber were induced and differentiated into mature adipocytes by MDI induction as described above; (3) the human APN recombinant adenovirus group (LPS+Ad-apM1), in which the 3T3-L1 cells were infected by human APN recombinant adenovirus with 100 of multiplicity of infection (MOI) as previously discussed [19]. After 48 h culturing, 3T3-L1 cells were cocultured with LPS-stimulated mature adipocytes and induced differentiation via MDI; (4) the Ad-apM1 and Ad-nnat-shRNA group (LPS+Ad-apM1+Ad-nnat-shRNA), in which the 3T3-L1 cells were infected by Ad-apM1 and Ad-nnat-shRNA with 100 of MOI, respectively. After 48 h culturing, 3T3-L1 cells were cocultured with LPS-stimulated mature adipocytes and induced differentiation via MDI; (5) the negative control (NC) group, in which the 3T3-L1 cells were infected by adenoviruses holding Ad-apM1 and empty plasmids with 100 of MOI, respectively. After 48 h culturing, 3T3-L1 cells were cocultured with LPS-stimulated mature adipocytes and induced differentiation via MDI. The time point and titer of adenovirus used in the current experiments were determined during preliminary experiments. The following experiments on 3T3-L1 cells in the inner chamber were performed at day 8.

### 2.5. Oil Red O Staining

The 3T3-L1 cells in the inner chamber of each group were washed with phosphate-buffered saline (PBS) three times, then fixated by 10% formalin for 1 h at room temperature, washed once with 60% isopropanol, and left to dry completely. 2 ml Oil Red O was added (Sigma-Aldrich; USA) for 2 h at 37°C, rinsed with 60% isopropanol once, and carefully washed with PBS four times at the end. Then the pictures were captured using an inverted microscope. What is more, Oil Red O was extracted with 100% isopropanol and determined spectrophotometrically at a wavelength of 490 nm via SpectraMax Paradigm (Molecular Devices, LLC) [20].

### 2.6. Real-Time Quantitative PCR Analysis

The total RNA of 3T3-L1 cells in the inner chamber was extracted by TRIzol reagent (Invitrogen, USA). Reverse transcription reactions were exerted with Transcriptor First Strand cDNA Synthesis Kit (Roche, Indianapolis, IN, USA). Premier Primer 5.0 software (Premier Biosoft International, Palo Alto, CA, USA) was used to design the oligonucleotide primer sequences as shown in Table 1. *β*-Actin was used as an internal control. With SYBR Green PCR Master Mix (Toyobo Life Science, Osaka, Japan), synthesized first-strand cDNA samples were subjected to PCR using ABI Prism 7700 Sequence Detector (Applied Biosystems. USA). Thermocycling conditions were as follows: 2 min at 95°C followed by 40 cycles of 15 sec at 95°C, 15 sec at 60°C, and 20 sec at 72°C, with a final extension step of 60°C for 30 min. A Dissociation curve was used to confirm the integrity of PCR products through 7500 software version 2.0.4 (Applied Biosystems; Thermo Fisher Scientific, Inc.). The threshold cycle (CT) values were determined, and the formula 2^-*ΔΔ*CT^ was used to calculate relative gene expression.

### 2.7. Assessment of Oxidative Stress

The 3T3-L1 cells in the inner chamber of each group were harvested and lysed by ultrasonication in the presence of a protease inhibitor. The supernatant was collected for analysis after centrifugation at 3,000 × g for 10 min. The levels of MDA, T-AOC, and SOD in the supernatant were measured using appropriate kits (Jiancheng Bioengineering Institute, CHN) following the manufacturer's instructions.

ROS was measured using the ROS assay kit (Jiancheng Bioengineering Institute, CHN). Cell suspension was obtained by tryptase digestion. 2′,7′-Dichlorofluorescein was used as a probe. The detection procedure was carried out in accordance with the instructions provided by the manufacturer.

### 2.8. Western Blotting Assay

Harvested cells in the inner chamber or ground EAT samples were lysed using Radio Immunoprecipitation Assay (RIPA; Solarbio, Beijing, China) lysis buffer, then centrifuged at 10,140 × g at 4°C for 15 min and harvested the supernatant. Bicinchoninic acid (BCA) protein assay kit (Beyotime, Haimen, China) was used to analysis total protein concentrations. Total protein (40 *μ*g) was diluted with sample buffer containing 100 mM dithiothreitol and heated in water bath at 98°C for 5 min, and then they were subjected to 10-15% sodium dodecyl sulfate polyacrylamide gel electrophoresis in a gel apparatus (Bio-Rad, USA) and transferred to PVDF membranes subsequently. The membranes were soaked in 5% nonfat dry milk for 2 h and incubated overnight at 4°C with the primary antibodies (1 : 500). After washing with TBS-T, the membranes were incubated with horseradish peroxidase-conjugated secondary antibody (1 : 2,000) shaking at room temperature for 1 h, then washed with TBS-T three times 20 min each. Lastly, the immune complexes were visualized via enhanced chemiluminescence (Tiangen Biotech Co., Ltd., Beijing, China) [21]. Final images were analyzed with Quantity One version 4.62 (Bio-Rad Laboratories, Inc.).

### 2.9. Statistical Analysis

Data are presented as the means ± standard error of the mean. SPSS version 20.0 (IBM Corp., Armonk, NY, USA) was used to perform the variance homogeneity test and one-way analysis of variance. The least significant difference method was used to compare the groups. *p* < 0.05 was considered to indicate a statistically significant difference.

## 3. Results

### 3.1. Effect of Nnat Knockdown on APN Promoting Preadipocyte Differentiation

**Figure 2:**
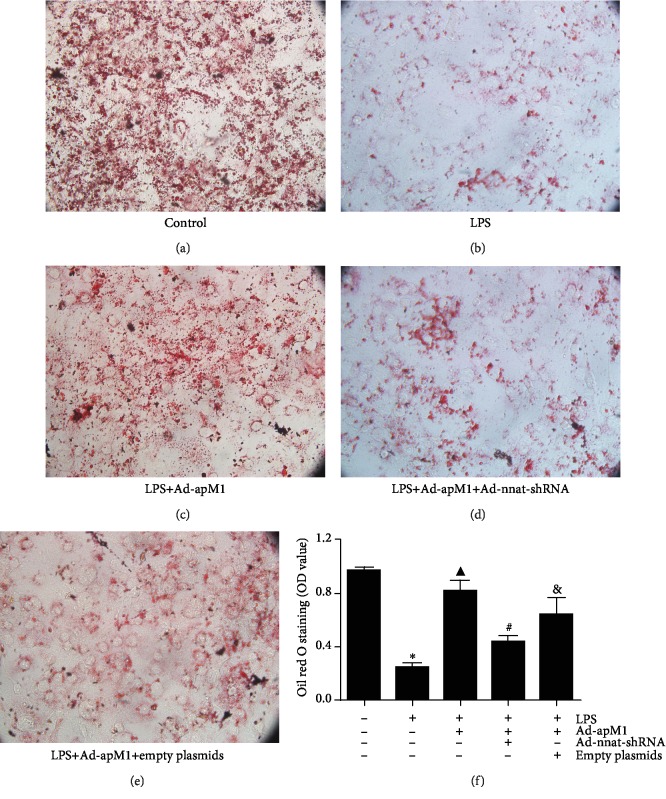
Effect of Nnat knockdown on APN promoting preadipocyte differentiation. (a–e) 3T3-L1 cells in each group stained by Oil Red O (magnification, ×40). (f) Oil Red O was extracted from cells with 100% isopropanol, and absorbance was determined spectrophotometrically at 450 nm. Data are presented as the mean ± standard error of the mean for six independent experiments. ^∗^*p* < 0.05 vs. the control group; ^▲^*p* < 0.05 vs. the LPS group; ^#^*p* < 0.05 vs. the LPS+Ad-apM1 group; ^&^*p* < 0.05 vs. the LPS+Ad-apM1+Ad-nnat-shRNA group.

Differentiation of preadipocytes was evaluated by Oil Red O staining. As shown in Figure 2, after 8 days, MDI-induced differentiation, cell volume, and the number of lipid droplets were obviously reduced, and lipid content significantly decreased in the LPS group when compared with the control groups (*p* < 0.05). APN overexpression could markedly promote differentiation of preadipocytes in an inflammatory environment (*p* < 0.05 vs. the LPS group). However, the effect of APN was significantly attenuated when the gene expression of Nnat was downregulated (*p* < 0.05), which suggests that Nnat is involved in the process of APN promoting preadipocyte differentiation.

### 3.2. Effect of Nnat Knockdown on APN Suppressing mRNA Expressions of Inflammatory Factors

**Figure 3:**
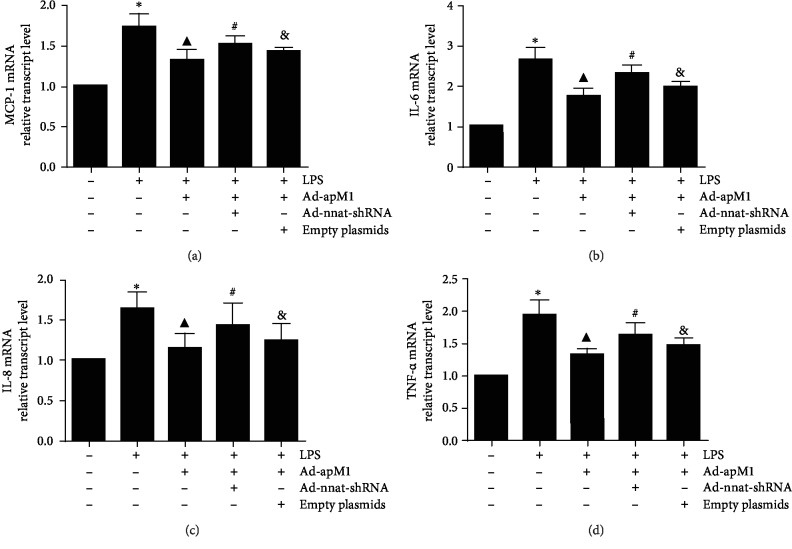
Effect of Nnat knockdown on APN suppressing mRNA expression of inflammatory factors. Relative mRNA expression levels of (a) MCP-1, (b) IL-6, (c) IL-8, and (d) TNF-*α* were detected using qPCR. Data are presented as the mean ± standard error of the mean for six independent experiments. ^∗^*p* < 0.05 vs. the control group; ^▲^*p* < 0.05 vs. the LPS group; ^#^*p* < 0.05 vs. the LPS+Ad-apM1 group; ^&^*p* < 0.05 vs. the LPS+Ad-apM1+Ad-nnat-shRNA group.

Expression levels of inflammatory factors including chemoattractant protein-1 (MCP-1), interleukin- (IL-) 6, IL-8, and tumor necrosis factor- (TNF-) *α* were detected by qPCR. As shown in Figure 3, compared with the control group, the mRNA expressions of MCP-1, IL-6, IL-8, or TNF-*α* were significantly increased in the group of LPS only (*p* < 0.05), while they were all significantly decreased under the overexpression of APN (*p* < 0.05 vs. the LPS only). Interestingly, APN's effects were attenuated in the LPS+Ad-apM1+Ad-nnat-shRNA group (*p* < 0.05, the LPS+Ad-apM1 group vs. the LPS+Ad-apM1+Ad-nnat-shRNA group).

### 3.3. Effect of Nnat Knockdown on APN Reducing Oxidative Stress Reaction

**Figure 4:**
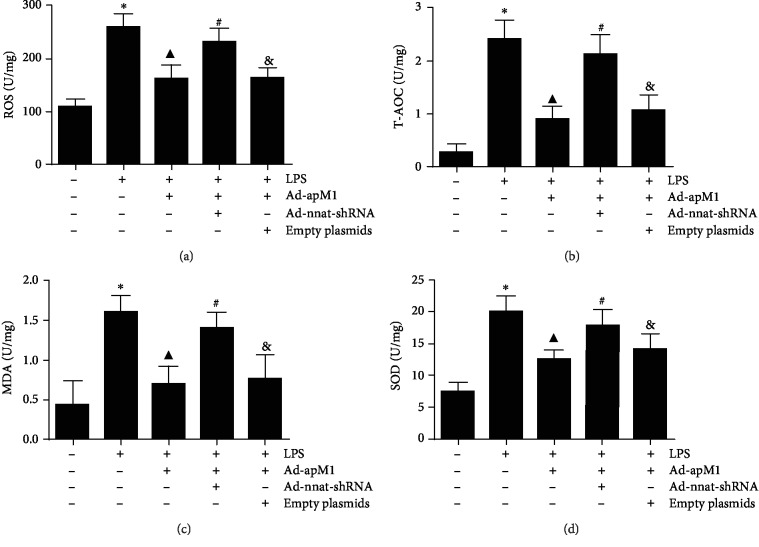
Effect of Nnat knockdown on APN reducing oxidative stress reaction. (a) The level of ROS in 3T3-L1 cells. (b) The level of T-AOC in 3T3-L1 cells. (c) The level of MDA in 3T3-L1 cells. (d) The level of SOD in 3T3-L1 cells. Data are presented as the mean ± standard error of the mean for six independent experiments. ^∗^*p* < 0.05 vs. the control group; ^▲^*p* < 0.05 vs. the LPS group; ^#^*p* < 0.05 vs. the LPS+Ad-apM1 group; ^&^*p* < 0.05 vs. the LPS+Ad-apM1+Ad-nnat-shRNA group.

As shown in Figure 4, ROS and MDA levels were significantly increased while T-AOC and SOD levels were significantly decreased in the LPS group compared with the control (*p* < 0.05). Overexpression of APN could markedly decrease ROS and MDA levels and increase T-AOC and SOD levels (*p* < 0.05), which were obviously reversed when Nnat was knocked down (*p* < 0.05, the LPS+Ad-apM1 group vs. the LPS+Ad-apM1+Ad-nnat-shRNA group).

### 3.4. Effects of Nnat Knockdown on Protein Expression Levels of Nnat and PPAR *γ* in each Group

**Figure 5:**
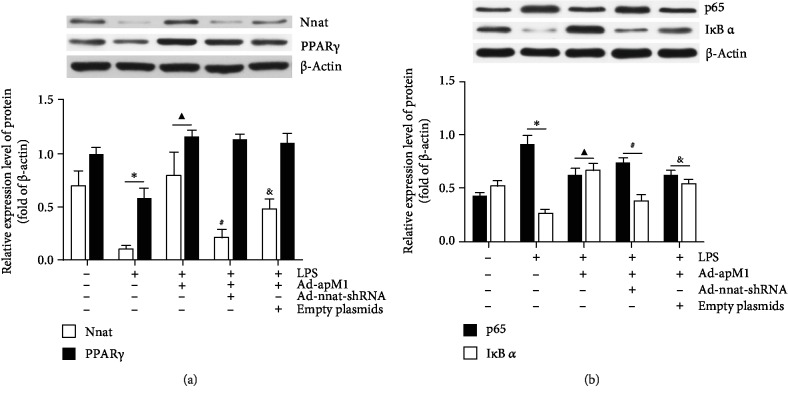
The protein expression levels of Nnat, PPAR *γ*, p65, and I*κ*B *α* in each experimental group. Cell lysates were collected and subjected to western blotting to examine the protein expression levels of Nnat, PPAR *γ*, p65, and I*κ*B *α*. (a) The protein expression levels of Nnat and PPAR *γ* in each experimental group. (b) The protein expression levels of p65 and I*κ*B *α* in each experimental group. Values were means ± S.E.M. for six individual experiments. ^∗^*p* < 0.05 vs. the control group; ^▲^*p* < 0.05 vs. the LPS group; ^#^*p* < 0.05 vs. the LPS+Ad-apM1 group; ^&^*p* < 0.05 vs. the LPS+Ad-apM1+Ad-nnat-shRNA group.

Western blotting was used to detect the protein expression levels of Nnat and PPAR *γ* in each group. As shown in Figure 5(a), protein expression levels of Nnat and PPAR *γ* were significantly downregulated in the LPS group (*p* < 0.05 vs. the control). Overexpression of APN could significantly increase the expression of Nnat and PPAR *γ* (*p* < 0.05 vs. the LPS only). When the Nnat gene expression was knockdown, the protein expression of Nnat was reversed obviously (*p* < 0.05 vs. the LPS+Ad-apM1 group), while PPAR *γ* had no significant change (*p* > 0.05 vs. the LPS+Ad-apM1 group).

### 3.5. Effects of Nnat Knockdown on Expression Levels of p65 and I*κ*B *α* in each Group

As shown in Figure 5(b), p65 protein expression level in 3T3-L1 cells was significantly increased in an inflammatory environment compared with the control (*p* < 0.05). However, APN overexpression significantly decreased the expression level of p65 (*p* < 0.05 vs. the LPS group). Conversely, the expression levels of p65 were markedly upregulated in the LPS+Ad-apM1+Ad-nnat-shRNA group (*p* < 0.05 vs. the LPS+Ad-apM1 group). Additionally, the LPS suppressed the I*κ*B *α* protein expression in the LPS group (*p* < 0.05 vs. the control), whereas its expression was upregulated with APN overexpression (*p* < 0.05 vs. the LPS group). However, the expression level of I*κ*B *α* was markedly downregulated in the LPS+Ad-apM1+Ad-nnat-shRNA group (*p* < 0.05 vs. the LPS+Ad-apM1 group).

## 4. Discussion

In the current study, we found that the beneficial effects of APN on promoting preadipocyte differentiation were through anti-inflammation and antioxidative stress, which was consistent with previous studies [17, 22]. However, the effects could be attenuated by knocking down the expression of Nnat. It indicates that Nnat is associated with the effects of APN on preadipocyte differentiation. In adult white adipose tissue and aortic endothelial cells, Nnat is highly expressed and may be involved in the pathogenesis of various human diseases [23, 24]. Suh et al. [25] have shown that Nnat could regulate preadipocyte differentiation via potentiation of cAMP-response element binding protein- (CREB-) mediated transcription of CCAAT/enhancer binding protein (C/EBP)b, C/EBPd, and C/EBPa by enhancing CREB phosphorylation through increasing the intracellular Ca^2+^ level. Therefore, we believe that APN promotes the differentiation of preadipocytes and accelerates adipocyte metabolism which may involve the Nnat pathway.

Inflammatory cytokines, including MCP-1, IL-6, IL-8, and TNF-*α*, which are secreted by adipose tissue, make major contributions to adipose tissue dysfunction and metabolic disorders [26, 27]. These dysfunctions and disorders are important causes of disease development, especially atherosclerosis [7]. Ka et al. [28] provided evidences showing that Nnat plays an anti-inflammation role in the adipose tissue via overexpression of Nnat in LPS-stimulated 3T3-L1 cells. In the present study, we observed that the expression of Nnat was upregulated with APN overexpression, and inflammatory factors made comebacks when the expression of Nnat was downregulated. It prompts that Nnat is involved in anti-inflammation effects of APN.

Mzhavia et al. [24] first demonstrated that Nnat expression could induce activation of nuclear factor kappa-B (NF-*κ*B) in endothelial cells. They found that Nnat expression promotes a gene expression signature in endothelial cells associated with increased inflammatory cytokines including TNF-*α*, Il-1*β*, and lL-6, endothelial cell adhesion molecules, and chemokine expression. In addition, NF-*κ*B can also participate in the oxidative stress process via regulating the production of ROS and affecting the levels of SOD and MDA through nicotinamide adenine dinucleotide phosphate (NADPH) oxidase [29]. Oxidative stress can affect adipose metabolism [30] and participate in the occurrence and development of many diseases, including atherosclerosis [22], obesity [31], hypertension [32], and diabetes [33]. We have detected the protein expression levels of p65 and I*κ*B *α*, the important members of NF-*κ*B family, and found that when the expression of Nnat protein was inhibited, the effects of APN on the downregulation of p65 and upregulation I*κ*B *α* were reversed, which suggested that Nnat mediates the NF-*κ*B signal pathway and then reduced the expression of inflammatory factors and oxidative stress reactions as a result. Taken together, we believe that the anti-inflammation and antioxidative stress effects of APN in adipocytes are through the Nnat/NF-*κ*B pathway.

What is more, we also detected the expression of PPAR *γ* in each group. PPAR *γ* is an essential regulator of adipogenesis. It is necessary and sufficient for differentiation of preadipocytes [34]. PPAR *γ* is known to operate in a cellular network with other transcription factors of the early B-cell factor (EBF), C/EBP, Kruppel-like factors (KLF), and interferon regulatory factor (IRF) families in regulating adipogenesis [35–37]. PPAR *γ*, the most highly expressed in white adipose tissue and has a crucial role in adipocyte metabolism, is the most significant transcription factor binding site for the key adipose tissue regulator in Nnat promoter sequences [13]. In the present study, the expression level of PPAR *γ* was reduced in LPS-induced 3T3-L1 cells, and APN could upregulate its expression as according to a previous study [17]. When Nnat was knocked down, no significant change was observed in the PPAR *γ* expression level. Therefore, we speculated that Nnat is regulated by PPAR *γ* to exert anti-inflammation and antioxidative stress and promoting adipocyte differentiation.

In conclusion, the present study demonstrated that APN could attenuate inflammation and oxidative stress in LPS-induced inhibition of preadipocyte differentiation maybe via activating the PPAR *γ*/Nnat/NF-*κ*B axis. What needs to be emphasized is that the inflammatory environment of EAT in the present study was simulated by LPS-stimulated 3T3-L1 mature adipocytes, which obviously differs from the actual situation in human EAT. Although there is still controversy about whether APN is beneficial to atherosclerosis, the exploration of APN in antiatherosclerosis is still of great significance [8, 9, 38]. Further researches will be conducted to explore the role of APN in improving atherosclerosis.

## Figures and Tables

**Table 1:** qPCR primers.

Gene title	Primer
*β*-Actin	Forward: TTACAGGAAGTCCCTCACCCTC Reverse: TCAGGGCATGGACGCGA
MCP-1	Forward: AACTGCATCTGCCCTAAGGT Reverse: ACTGTCACACTGGTCACTCC
IL-6	Forward: ACAAAGCCAGAGTCCTTCAGAG Reverse: GTGACTCCAGCTTATCTCTTGGT
IL-8	Forward: GCACTTGGGAAGTTAACGCA Reverse: GCACTTGGGAAGTTAACGCA
TNF-*α*	Forward: AGCCGATGGGTTGTACCTTG Reverse: ATAGCAAATCGGCTGACGGT

## Data Availability

The scientific and statistical data used to support the findings of this study are included within the article. Requests for access to these data should be addressed to Wenkai Yang at yangwenkai111@163.com.
